# Mental health status and work environment among workers in small- and medium-sized enterprises in Guangdong, China-a cross-sectional survey

**DOI:** 10.1186/1471-2458-14-1162

**Published:** 2014-11-12

**Authors:** Zhi Zeng, Yan Guo, Liming Lu, Lu Han, Wen Chen, Li Ling

**Affiliations:** Sun Yat-sen Center for Migrant Health Policy, School of Public Health, SunYat-sen University, Guangzhou, 510405 China; Maternal and Child Health Hospital of Hunan Province, Changsha, 410008 China

**Keywords:** Chinese workers, Mental health, Psychological well-being, WHO (five) well-being index, Small- and medium-sized enterprises (SMEs), Job demand-control (JDC) model, Job demand-control-support (JDCS) model

## Abstract

**Background:**

Small- and medium-sized enterprises (SMEs) generate nearly 80% of the jobs in China, but the dangerous work environment often found in these enterprises poses a major concern for public health. Psychosocial pressure and mental health problems among the workers are also common in SMEs. However, mental health of workers in SMEs is largely neglected in occupational health research and practice in China. The purpose of this study is to assess mental health of the workers and to explore the associations between physical and psychosocial work environment and workers’ mental health in SMEs in South China.

**Methods:**

Data were collected in 2012 through a cross-sectional survey among 1200 workers working in small- and medium-sized enterprises (SMEs) in Guangdong, China. Mental health was measured by psychological well-being in the current study. Job Demand-Control-Support (JDCS) model was used as a theoretical framework to examine the psychosocial factors associated with workers’ psychological well-being. Data were analyzed using SPSS 20.0 and analysis was performed using bivariate analyses and multivariate logistic regression.

**Results:**

About three in ten workers (35.3%) in the sample had poor psychological well-being. Those who were men, younger in age, or migrant workers had worse psychological outcome in bivariate analyses. After controlling for individual variables (gender, age, marital status, and household registration), we found that longer weekly work hours (OR = 1.30, 95% CI: 1.13 ~ 1.50), more exposure to hazardous work environment (OR = 1.26, 95% CI: 1.10 ~ 1.44), higher job demands (OR = 1.29, 95% CI: 1.12 ~ 1.49), and lower job autonomy (OR = 0.70, 95% CI: 0.60 ~ 0.81) were significant associated with worse psychological well-being. The results were consistent with predictions of the JDCS model.

**Conclusions:**

The results indicate that the JDCS model is a useful framework in predicting psychological well-being among Chinese workers in SMEs. Future mental health promotion should focus on young migrant male workers as they appear to be most vulnerable in their psychological well-being. Both physical and psychosocial aspects of the work environment should be taken into account in policy making to prevent mental disorder and promote psychological well-being among workers in SMEs.

## Background

China has experienced dramatic industrialization, urbanization, and economic growth over the last three decades. Small- and medium-sized enterprises (SMEs) play a crucial role in the Chinese economy, providing about 80% of the jobs in urban China [1]. Compared to large enterprises, SMEs tend to be less regulated in safe work environment. In addition, employees in SMEs usually have low wages and lack of effective long-term occupational health services [2]. Rural-to-urban migrants consist of the majority of the employees in SMEs who migrate from less developed areas to more developed areas in China [3]. It was estimated that the number of migrant workers had reached 263 million (19.4% of the total population) in 2012 [4]. Compared to local residents, mental health problems and suicides are more likely to occur in these migrants [5]. Studies have shown that mental health of the workers in SMEs is worse than both the general population and those in large enterprises [6, 7]. However, mental health of the workers in SMEs is largely neglected in existing occupational health research and practice in China [8].

It is estimated that the burden of mental illness will account for 1/4 of the total disease burden in 2020 globally [9]. Mental health problems are common in the Chinese working population. Depression is one of the most disabling of mental disorders. According to the Chinese Health Education Center Survey, about 50% of the 13,177 workers sampled in 6 provinces or municipalities had symptoms of depression [10]. The definition of mental health is not just the occurrence of mental disorder, but also a state of absence of well-being, from the perspective of primary prevention [11]. Literature suggests that decreased well-being is the first sign of distress [12, 13].

Working conditions are key to understanding psychological well-being of workers who are frequently exposed to hazardous environment. Both physical and psychosocial work environment are important. According to previous studies, shift work, long work hours, hazardous work environment (e.g., exposure to noise or toxic chemicals) might be potential risk factors for poor mental health of the Chinese workers [14–17]. Research has also shown a close relationship between psychosocial work-related factors and mental health of workers [18]. Such relationship, however, is largely neglected in the existing literature on workers in China.

Over the past several decades, one of the most influential models in examining the relationship between work environment and mental health is the Job Demand-Control-Support (JDCS) model. The JDCS model identifies three critical aspects at work: job demands, job autonomy and worksite support [19]. A recent meta-analysis reveals that high job demands and low job autonomy are risk factors for poor mental health and that the psychosocial aspect of the work environment is important for workers’ mental health [20]. However, there are few empirical studies utilizing the JDCS model and considering both physical and psychosocial work-related factors on the mental health of the SMEs workers in China.

The purposes of the current study are to assess the mental health status of the SMEs workers, and to explore the associated work-related factors (both physical and psychosocial) with the mental health status of the SMEs workers in South China.

## Methods

### Study setting

This cross-sectional study was conducted between September and October in 2012 in Guangdong Province, China. A two-stage stratified random sampling method was carried out. First, two developed cities (Guangzhou and Foshan) and two less-developed cities (Zhaoqing and Qingyuan) in Guangdong Province were selected. Second, based on the final sample size requirement (see next section for detail), we randomly selected SMEs in shoe making, electronics, and plastics industries from each city by applying computer-generated random numbers from the list of enterprises in these cities. The reason to choose industries in shoe making, electronics, and plastics is that workers in these industries were exposed to the same occupational hazard, organic solvent, which causes about 50% of all cases related to occupational diseases in Guangdong Province [21]. The county Centers for Disease Control and Prevention (CDC) (or occupational health institute) assisted in the selection process and encouraged the selected local enterprises to participate in the study. Finally, front-line workers in assembly lines were recruited from the sampled SMEs for participation.

### Sample size estimation

According to literature review, the rate of depression among occupational group was 50% in China [10]. Assuming 95% confidence level and an absolute sampling error of 4%, a minimum of 600 workers are needed. From literature and our previous experience, taking into account of the 70% of response rate and 80% of completion rate, the needed sample size was 1071.

### Study Subjects

The recruitment criteria for workers included: 1) front-line workers in assembly lines who were exposed to occupational hazards (noise, or toxic chemicals); 2) workers who had resided in the study area for at least six months. Exclusion criteria included team leaders or management personnel who differ in their work environmental exposure [22]. Workers completed the questionnaire individually. Trained investigators provided assistance to participants whenever needed. Each questionnaire was checked by two investigators for missing data or logical inconsistency. It typically took about 20 minutes to finish the survey. A small monetary incentive (RMB 20, equal USD 3.26) was provided for participants upon their completion of the survey. Finally, data from 1200 workers (response rate, 90%) in 10 enterprises were analyzed.

### Ethics statement

The study was approved by the Institutional Review Board (IRB) of the School of Public Health, Sun Yat-sen University in China. Written informed consents were obtained from all study participants.

### Measurements

#### WHO well-being scale

We employed psychological well-being to examine mental health status of workers in SMEs in China. The World Health Organization (WHO) (five) Well-Being Index, 1998 version (WHO-5) [23] was designed for measuring psychological well-being. It is the short form (five items) of the earlier versions of this instrument [24]. The WHO-5 is recommended by the WHO as the first step in a two-stage screening process for depression and should be followed by a clinician interview [25]. The diagnostic validity of the WHO-5 in the screening of depression is good [Area Under the Curve (AUC) value = 0.88], as indicated by several studies [26–28].

WHO-5 is a uni-dimensional measure that asks the feelings of participants in the previous two weeks and contains five positively-worded items: "I have been cheerful and in good spirits"; "I have been calm and relaxed"; "I have been active and vigorous"; "When I wake up, I feel fleshed and rested"; and "My daily life is filled with things that interest me". The options of the answers regarding their feelings are on a 6-point likert scale ranging from 0 (not present at all) to 5 (constantly present). The possible total scores vary from 0 to 25, with higher scores indicating better well-being. Scores of 13 and below indicate poor well-being and warrant follow-up diagnostic procedures [26, 29, 30].

#### Physical work conditions

Physical work conditions were measured by whether workers had employment contract (yes/no) and shift work schedule (yes/no), how many work hours per week, and physical work environment which was assessed by two questions:" How often are you exposed to disturbing noise?" "How often are you exposed to excessive toxic chemicals?" Responses for the two exposure questions were on a five-point scale ranging from 1(not at all) to 5 (almost all the time).

#### Psychosocial work conditions

According to the Job Demand-Control-Support (JDCS) model [19], and Chinese (Mainland) Version of Job Content Questionnaire [31], psychosocial work conditions were measured by three dimensions, including job demands, job autonomy, and worksite support. Job demands referred to work load, and had been operationalized mainly in terms of time pressure and role conflict. Job autonomy referred to a person’s ability to control his or her work activities. Worksite support included support from supervisors and coworkers. Responses were on a five-point scale ranging from 1 (strongly disagree) to 5 (strongly agree). Please see the details of the JDCS model construct items in Table 1.

**Table 1:** **The JDCS Model Construct Items**

Construct	Item	Item text	Range	Cronbach’s Alpha
Job demands	1	My job requires working very fast.	2 ~ 10	0.70
	2	My job requires working very hard.		
Job autonomy	1	I have enough resources to manage my job.	3 ~ 15	0.73
	2	At my workplace I dare to speak my minds.		
	3	I have the freedom to decide how my tasks are to be carried out.		
Worksite support	1	I get along well with my supervisors.	2 ~ 10	0.61
	2	I get along well with my coworkers.		

#### Individual characteristics

Participants also provided individual characteristics on gender, age, educational attainment (primary school or lower, junior high school, senior high school, college or higher), individual monthly income, household registration (permanent residents or migrant workers).

#### Statistical analyses

A database was constructed using Epidata 3.0 and statistical analyses were performed using SPSS version 20.0. First, as part of a test of reliability, internal consistency was examined using Cronbach’s alpha. WHO (five) Well-Being Index, extent of exposure to hazardous environment, job demands, job autonomy, and worksite support demonstrated a reliability alpha coefficient of 0.91, 0.70, 0.70, 0.73, 0.61, respectively, suggesting that all of the measures had acceptable to satisfactory internal reliability [32].

The WHO (five) Well Being Index scale scores were calculated according to the established algorithms with higher scores indicating better mental health status. Well-being scores were categorized into two categories (1 ~ 13/14 ~ 25). The distribution of individual characteristics and work-related factors were illustrated by numbers and percentages, means and standard deviations as appropriate. Bivariate analyses were employed to analyze the associations between factors and mental health. Multivariate logistic regressions were used to simultaneously identify work-related variables associated with mental health, while adjusting for individual characters significant in bivariate analyses at *P* < 0.05. Odds ratios (ORs) with 95% confidence interval (CI) were presented. Two-sided tests of significance and confidence intervals were based on the 0.05 level.

## Results

### Characteristics of the study population

Table 2 presents individual and work-related characteristics of the workers as well as their mental health status. Most of the workers were male (54.4%), being married or co-habiting (60.4%). The average age was 31.1 years. Majority of the participants were migrant workers (75.7%) and had signed employment contract (92.8%); about two fifths (39.1%) worked on a shift work schedule, with average work hours of 55.4 per week and monthly income of 2431RMB (USD 396) (The average monthly wage of workers was 3762 RMB (USD 617) in urban areas of Guangdong Province in 2012) [33]. About one third (35.3%) of them reported poor psychological well-being in the past two weeks.

**Table 2:** **Individual and work-related characteristics and mental health of the study population (** ***N*** **= 1200)**

Variables	***N***(%)
**Individual characteristics**	
Age *(years) (Mean ± SD)*	*31.1 ± 9.4*
Gender	
Men	653(54.4)
Women	547(45.6)
Marital status	
Married or co-habiting	727(60.5)
Single	464(38.7)
Divorced	9(0.8)
Education level	
Primary school or lower	79(6.6)
Junior high school	587(48.7)
Senior high school	443(37.1)
College or higher	91(7.6)
Individual monthly income *(yuan) (Mean ± SD)*	*2431 ± 937*
Household registration	
Permanent resident workers	293(24.3)
Migrant workers	907(75.7)
**Physical work conditions**	
Employment contract	
Yes	1114(92.8)
No	86(7.2)
Shift work schedule	
Yes	466(39.1)
No	734(60.9)
Work hours per week *(hours) (Mean ± SD)*	*55.4 ± 8.1*
Extent of exposure to hazardous environment *(Mean ± SD)*	*5.5 ± 2.4*
**Psychosocial work conditions**	
Job demands *(Mean ± SD)*	*4.4 ± 1.5*
Job autonomy *(Mean ± SD)*	*10.5 ± 2.3*
Worksite support *(Mean ± SD)*	*7.5 ± 1.1*
**Mental health**	
Psychological well-being	
0 ~ 13	424(35.3)
14 ~ 25	776(64.7)

### Comparative analyses

Summaries of the bivariate analyses are presented in Table 3. Those who were men, younger in age, or migrant workers were more likely to have worse psychological well-being than their counterparts. Educational level, individual monthly income, shift work schedule were not significantly associated with psychological well-being. More exposure to hazardous work environment, higher job demands, and lower job autonomy were risk factors for psychological well-being. Workers with longer work hours per week had worse psychological well-being, and those who signed a contract with enterprises had better psychological well-being.

**Table 3:** **Bivariate analyses showing association of individual and work-related factors with mental health status of workers in SMEs (N = 1200)**

Variables	Poor psychological well-being	
	0 ~ 13	14 ~ 25
	(***N*** = 424, 35.3%)	(***N*** = 776, 64.7%)
**Individual characteristics**		
Age *(years) (Mean ± SD)*	28.6 ± 8.7**	*32.4 ± 9.6*
Gender		
Men	265(40.2)**	388(59.8)
Women	159(29.6)	388(70.4)
Marital status		
Married/co-habiting	201(28.3)**	524(71.7)
Single	220(46.3)	244(53.7)
Divorced	3(33.3)	6(66.7)
Educational level		
Primary school or lower	25(32.5)	54(67.5)
Junior high school	202(33.9)	385(66.1)
Senior middle school	163(37.1)	280(62.9)
College or higher	34(37.4)	57(62.6)
Individual monthly income *(yuan) (Mean ± SD)*	*2350 ± 855*	*2462 ± 826*
Household registration		
Permanent resident workers	84(29.0)**	209(71.0)
Migrant workers	340(37.4)	567(62.6)
**Physical work conditions**		
Employment contract		
Yes	386(34.5)*	728(65.5)
No	38(45.7)	48(54.3)
Shift work schedule		
Yes	179(38.5)	287(61.5)
No	245(33.3)	489(66.7)
Work hours per week *(hours) (Mean ± SD)*	56.8 ± 7.9**	*54.6 ± 8.0*
Extent of exposure to hazardous environment *(Mean ± SD)*	5.8 ± 2.3**	*5.3 ± 2.3*
**Psychosocial work conditions**		
Job demands *(Mean ± SD)*	4.8 ± 1.4**	*4.2 ± 1.5*
Job autonomy *(Mean ± SD)*	9.8 ± 2.4**	*10.9 ± 2.2*
Worksite support *(Mean ± SD)*	*7.4 ± 1.1*	*7.5 ± 1.0*

*P < 0.05, **P < 0.01.

### Multivariate logistic regression

Table 4 presents the associated factors with mental health of participants in multivariate logistic analyses. Dependent variable was poor psychological well-being (0 ~ 13 = 1, 14 ~ 25 = 0). After controlling for individual characters (gender, age, marital status, and household registration) significant in bivariate analyses at *P* < 0.05, longer weekly work hours (OR = 1.30, 95% CI: 1.13 ~ 1.50), more exposure to hazardous work environment (OR = 1.26, 95% CI: 1.10 ~ 1.44) were significantly and negatively associated with poor psychological well-being. Higher job demands (OR = 1.29, 95% CI: 1.12 ~ 1.49), and lower job autonomy (OR = 0.70, 95% CI: 0.60 ~ 0.81) were significantly and negatively associated with poor psychological well-being. The results were consistent with the JDCS model as the model predicts psychosocial factors associated with mental health status of workers in SMEs [19].

**Table 4:** **Multivariate logistic regression analyses showing association of individual and work-related factors with mental health status of workers in SMEs (N = 1200)**

Variables	Poor psychological well-being (0 ~ 13 = 1/14 ~ 25 = 0)
	OR (95 %CI)
**Individual characteristics**	
Age *(years)*	0.74 (0.61 ~ 0.89)**
Gender	
Men *(reference)*	
Women	0.61 (0.46 ~ 0.82)**
Marital status	
Married/co-habiting *(reference)*	
Single	0.77 (0.54 ~ 1.11)
Divorced	1.18 (0.28 ~ 5.09)
Household registration	
Permanent resident workers *(reference)*	
Migrant workers	1.36 (1.01-1.85)*
**Physical work conditions**	
Employment contract	
Yes *(reference)*	
No	1.04 (0.62-1.73)
Shift work	
No *(reference)*	
Yes	0.86 (0.63-1.17)
Work hours per week	1.30 (1.13-1.50)***
Extent of exposure to hazardous environment	1.26 (1.10-1.44)***
**Psychosocial work conditions**	
Job demands	1.29 (1.12-1.49)**
Job autonomy	0.70 (0.60-0.81)***
Worksite support	0.97 (0.85-1.11)

*P < 0.05, **P < 0.01, ***P < 0.001.

Adjusting for individual characters (gender, age, marital status, and household registration) significant in bivariate analyses at *P* < 0.05.

## Discussion

The current study intended to assess mental health of workers in SMEs in Guangdong, China and to explore the related physical and psychosocial factors of the work environment. 35.3% of the workers were found poor in their psychological well-being status. Those who were men, younger in age, or migrant workers were more likely to have worse psychological well-being. Our study found significant associations between physical work-related factors (i.e., weekly work hours, extent of exposure to hazardous work environment) and mental health. In addition, we also found significant associations between psychosocial work factors (i.e., job demands and job autonomy) and mental health among workers in SMEs, guided by the Job Demand-Control-Support (JDCS) model [19].

Results of the current study are consistent with the predictions of the JDCS model (higher job demands and lower job autonomy were significantly and negatively associated with mental health). Such model has been widely applied to mental health and psychological well-being research [34, 35]. The current study is the first effort to apply the JDCS model to examine the mental health of Chinese workers in SMEs. Results of the current study indicate that the JDCS model is a useful framework predicting mental health among workers in SMEs in South China.

Even though the New Labor Law was passed in 2008 in China that regulated no more than 40 hours a week among workers, such regulation was not strictly enforced. In the current study, the average weekly work hours were 55.4 hours. Previous study suggests a negative association between work hours and workers’ mental status, with odds ratio being 2-4 for depression for those working more than 40 hours per week [36]. In our study, work hours is also a significant indicator for workers’ mental health. All the findings indicate the importance of reinforcing the regulations of the New Labor Law on maximum work hours for factory workers. Similar to other studies, results show that more exposure to hazardous work environment increases the risk of mental health problems among Chinese workers in SMEs [7, 37]. Workers exposed to physical hazard (e.g., noise) had relatively higher risks of developing mental health problems than others without such exposure [38].

Workers’ psychosocial well-being is not only associated with physical work environment (e.g., work hours, dangerous work environment), but also with psychosocial work environment. In the present study, high job demands and low job autonomy were associated with poor psychological well-being, which is consistent with the prediction of the Demand-Control model (JDC) and other studies [19, 39]. The French "GAZEL Study" found that compared to low levels of job demands, high levels of job demands increased the odds ratios for poor mental health by 1.8 for men and 1.4 for women; compared to high job autonomy, odds ratio for low job autonomy was 1.4 for both genders [40, 41]. However, we didn’t find significant association between worksite social support and mental health. Similarly, in van der Doef’s review of 232 studies on the Job Demand-Control-Support model and psychological well-being [42], 135 (58%) studies did not find statistically significant relationship between psychological well-being and worksite social support. Therefore, in order to improve workers’ psychological well-being, it may be an effective strategy to reduce job demands and increase workers’ job autonomy in workplace.

Previous study found that the depressive rates of the Chinese working population in mild, moderate, and severe status were 25.6%, 23.5%, and 1.52%, respectively [9]. Our study also shows high prevalence of poor psychological well-being (35.3%) in workers in SMEs. Besides researches that reports higher psychological distress levels among women [28, 43]. There are also literatures reporting men more likely to have worse psychological well-being [44, 45] which is similar to our study. In China, men are mainly responsible for matters outside of the home (which often means source of income), while women are mainly responsible for matters inside of the home (which often means chores at home). Compared with women, men have to confront more social and economic pressure, and prefer to face difficulties alone rather than share the burden. Consistent with previous findings, workers who are older are better in their mental health status as increased knowledge and experiences in work and life may help them better accustomed to environment [44, 45]. Age, in our study, is also associated with increased job autonomy and decreased job demands which may contribute to better health status among older workers [46]. Psychosocial well-being of migrant workers, as indicated in our study and previous studies, was worse than permanent resident workers [47]. Due to changes in living environment and reduced social network and support, migrant workers often experience more mental health problems than the local residents [47]. Future mental health promotion among factory workers needs to target particularly on migrant workers in SMEs as they are a most vulnerable population subject to hazard working environment.

There are several limitations of this study that should be acknowledged. First, the cross-sectional research design in the current study does not allow causal analyses, only associations. It is plausible that poor mental health may have preceded hazardous work environment (i.e., reverse causation). Associations between physical and psychosocial work-related factors and poor mental health should be further explored in longitudinal studies among SMEs workers in China. Second, we didn’t have objective measurements of work environment. All measurements are self-report which may subject to recall bias or socially desired preferences. Third, a wide range of factors may be related to mental health of workers, our study mainly included work-related factors and individual social demographic factors whereas other important factors (e.g., family relationship, social support) that may potentially influence mental health are not included. Fourth, we used a few key indicators (not a complete scale) to determine work-related factors based on the Job Demand-Control-Support (JDCS) model, and Chinese Version of Job Content Questionnaire. Even though all the Cronbach’s alpha of work-related factors is above 0.6 in our study, the validity of using such scale still needs to be further confirmed.

## Conclusions

Our study is one of the first to assess psychological well-being and examine the associated work environment factors among workers in SMEs in China. The results indicate that the JDCS model is a useful framework in examining psychological well-being among Chinese workers in SMEs. The primary policy implication is that young migrant male workers are a key population for mental health promotion. Both physical and psychosocial work environment should be taken into account in policy making to prevent mental illness and promote psychological well-being among workers in SMEs in China.

## Authors’ information

Zhi Zeng and Yan Guo: co-first authors.
